# A bibliometric analysis of the development trend of Tuina research: Based on the Web of Science (WOS) platform

**DOI:** 10.1097/MD.0000000000041070

**Published:** 2025-01-17

**Authors:** Lin Gao, Suyu Chen, Tianlei Gao, Liuying Yang, Xiantao Tai

**Affiliations:** a First Clinical Medical College, Yunnan University of Chinese Medicine, Kunming, China; b Second Clinical Medical College, Yunnan University of Chinese Medicine, Kunming, China.

**Keywords:** bibliometric analysis, Chinese massage, research review, Tuina

## Abstract

**Background:**

Tuina has been proven to significantly relieve symptoms, demonstrating its clinical value. With the growth of related research, a comprehensive review is urgently needed to promote its development. This study aims to clarify the collaboration and current research status in the field of Tuina research using bibliometric analysis, and to discuss future directions.

**Methods:**

On February 4, 2023, the Web of Science Core Collection (WoSCC) database was searched using the keywords “tuina,” “tui-na,” “tui na,” and “Chinese massage” to collect literature related to Tuina from its inception until December 31, 2022. The collected literature included all types of research articles and reviews. VOSviewer, CiteSpace, Pajek, and Scimago Graphica were utilized to perform a visual analysis of annual publication volume, as well as publication volume and collaboration networks of different countries, institutions, and authors, along with journal publication volume and keyword network analysis.

**Results:**

A total of 761 publications were included in the analysis, with the total number of papers showed an increasing trend over time. The countries and institutions with the most publications were China and Shanghai University of Traditional Chinese Medicine, respectively. The authors who contributed the most were Min Fang, Qingguang Zhu, Zhiwei Wu, Lingjun Kong and Yufeng Wang. The most widely published journal in the field was the *Journal of Acupuncture and Tuina Science*. The clinical efficacy evaluation of Tuina therapy for musculoskeletal and spinal diseases, apoplexy sequelae, chronic diseases, and pediatric diseases were research hotspots and developing trends in this field.

**Conclusion:**

The research on Tuina has been increasing year by year. Currently, the focus of Tuina research lies in clinical studies, including the treatment of skeletal muscle and spinal diseases, stroke sequelae, chronic diseases, and pediatric illnesses, with pediatric Tuina emerging as a hot topic of research. Basic research in this field is relatively scarce, and the mechanisms of action of this therapy have not yet been fully elucidated. Future efforts in this area should aim to strengthen basic research and promote cooperation among international institutions.

## 1. Introduction

Acupuncture and Tuina are the most influential disciplines of Traditional Chinese Medicine (TCM) in the world. Tuina is a noninvasive and comfortable therapy based on the theory of viscera and meridians of TCM and involves distinct and skillful operation. In recent years, Tuina has made certain progress in international research. Tuina therapy applies pressure on the skin, muscles, and bones to relax muscles, enhance local metabolism, and correct skeletal misalignments. As a consequence, it can be used to effectively treat musculoskeletal disorders like lumbar disc herniation,^[1]^ low back pain,^[2,3]^ and chronic neck pain.^[4]^ In addition to its beneficial effects on pathological structures, there is evidence that Tuina techniques also regulate the central nervous system and endocrine systems. For example, by intervening in the transmission and processing of pain signals in the central nervous system, Tuina can achieve analgesic effects.^[5]^ Furthermore, Tuina can influence the activation of brain regions related to emotions, cognition, and sensory perception.^[3]^ It has also been shown that the application of Tuina therapy modulates the functionality of the digestive system by regulating the levels of corticosteroids, dopamine, and insulin.^[6]^ Tuina therapy has therefore been used in clinical settings to manage acute diarrhea.^[7]^ Taken together, these findings suggest that Tuina may have broad prospects for international research studies and future clinical applications.

Acupuncture, moxibustion, and Tuina are traditional Chinese medicine therapies that are widely used as complementary and alternative medical treatments in clinical practice. In recent years, bibliometric analysis methods have been extensively applied to evaluate the research status and trends of these therapies, such as acupuncture,^[8]^ moxibustion,^[9]^ and Qigong.^[10]^ With the increasing number of related research publications, the potential of Tuina therapy is gaining recognition. A bibliometric analysis of acupoint massage revealed the progress of acupoint massage research over the past 20 years, indicating that acupoint massage, as a part of Tuina therapy, has already seen significant application in clinical care.^[11]^ So, what about the research situation of Tuina therapy? Therefore, this study aims to use bibliometric analysis to scientifically assess the quality and impact of publications on Tuina research topics. Our goal is to outline the historical progress, current research status, and future development trends in this field, providing valuable reference information for scholars and clinicians engaged in scientific research and clinical practice.

## 2. Methods

All data were obtained from the Web of Science Core Collection (WoSCC) database on February 4, 2023. The search formula was as follows: TS= (“Tuina”) or (“Tui-Na”) or (“Tui Na”) or (“Chinese massage”). The time range of the search was set from the establishment of the database to December 31, 2022. The titles and abstracts were read independently by 2 researchers and reviewed by a third researcher to ensure that they conformed to scientific research literature related to Tuina. The inclusion criteria for literature retrieval were articles and reviews focused on Tuina, including but not limited to clinical studies, basic research, systematic evaluations, etc., restricted to English-language papers only. The following types of literature were excluded: editorial materials, letters, corrections, conference abstracts, book reviews, and news items.

The included literature was exported from the WoSCC database in text format, which contained the title, publication time, author, country, journal, and keywords of the article. These data were visualized and analyzed by three researchers using CiteSpace (https://citespace.podia.com/), VOSviewer (https://www.vosviewer.com/), Pajek (http://mrvar.fdv.uni-lj.si/pajek/), and Scimago Graphica (https://www.graphica.app/) to tabulate the statistics and draw visual analytical graphs.

VOSviewer is used to analyze the publication volume and collaboration networks of countries, institutions, and authors, as well as keyword clustering networks. Combined with Pajek and Scimago Graphica, this helped to draw a country cooperative map and network map. The parameter settings were as follows: The node types were “country,” “author,” “institution,” and “keyword” based on different types of analysis, with the counting method set as “Full counting.”

CiteSpace was used to analyze the burst keywords and draw the overlay map. The parameter settings were as follows: The time range was set to 1990 to 2022, the time slice was set to 1 year, and the node type was “keyword,” with a g-index k value of 25.

## 3. Results

### 3.1. Literature search and inclusion

**Figure 1.**
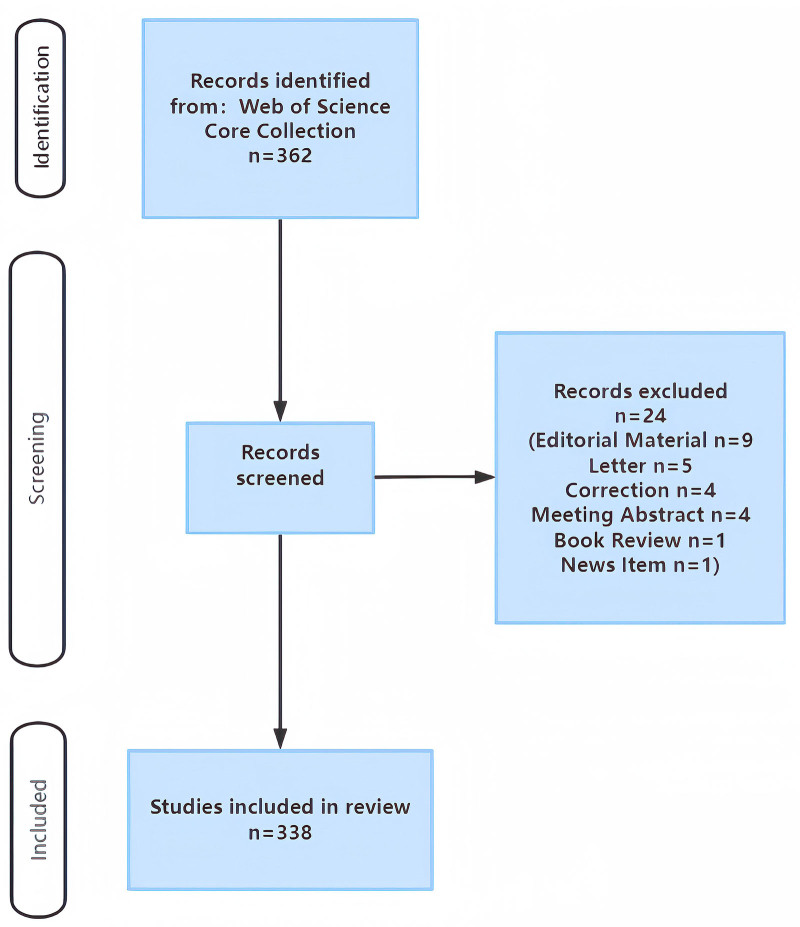
Flow chart for the search and selection strategy of the study.

A total of 817 articles were retrieved from the WoSCC database. After excluding documents other than articles and reviews, 762 publications remained for further screening by 2 researchers, resulting in the inclusion of 761 articles (Fig. 1). The screening results of the 2 researchers were consistent (Kappa = 1), as shown in Table 1.

**Table 1:** Kappa consistency test of inter-rater agreement on literature screening by 2 researchers

Paired items	Kappa value	Standard error	*z*	*P*
Researcher 1 paired with researcher 2	1	0.1	10	0.000***

*Indicates a significance level of 10%.

**Indicates a significance level of 5%.

*** Indicates a significance level of 1%.

### 3.2. The number and annual trend of published literature

**Figure 2.**
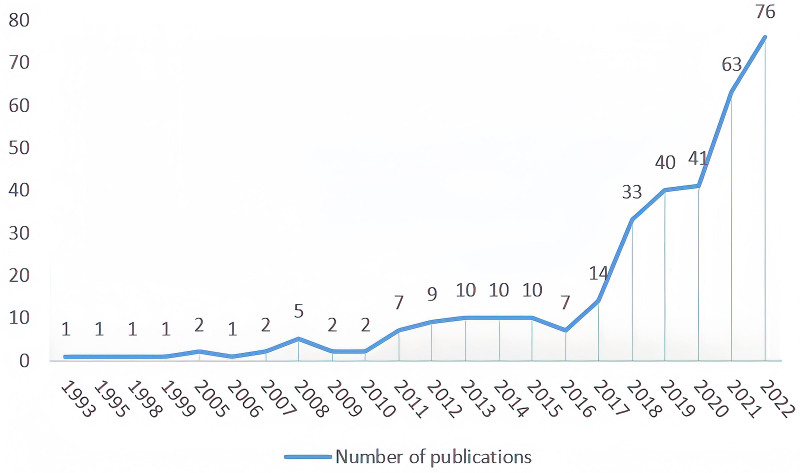
The annual number of publications related to Tuina (1993–2022).

As shown in Figure 2, although the number of published literature on Tuina fluctuated to a certain extent over the past 30 years, the overall number of papers showed an increasing trend year by year. The first paper on Tuina was published in 1993. In this article, the author reviewed the development history and relationship of acupuncture, Tuina, and Chinese herbal medicine and discussed whether students in all American acupuncture schools should be required to learn these three disciplines and prove their abilities through examination.^[12]^ Since 2016, research outputs in this field have shown a trend of rapid growth, with the number of publications increasing to 76 by 2022.

### 3.3. Distribution of countries

**Figure 3.**
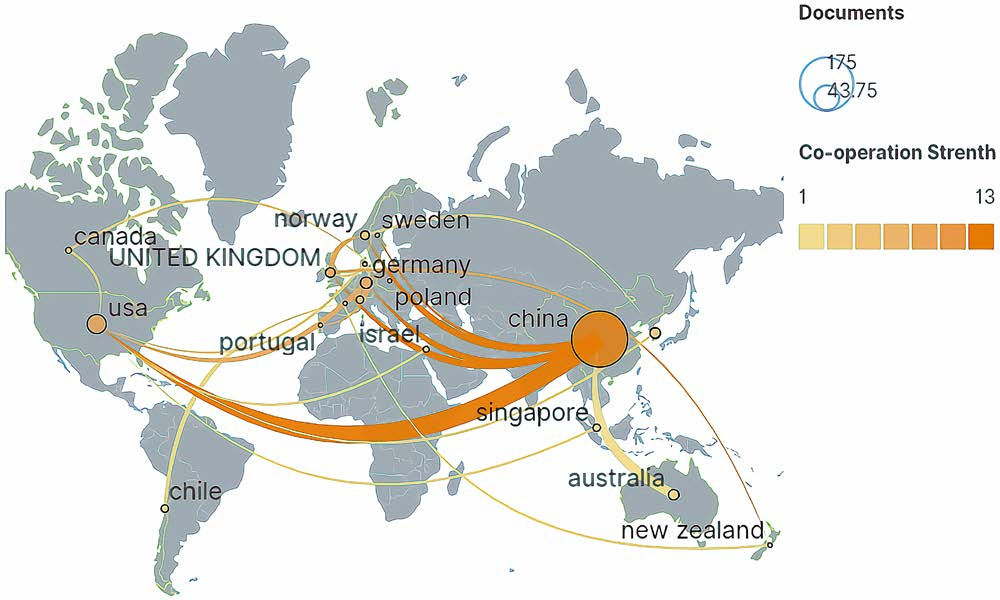
The collaboration network of countries researching Tuina (1993–2022).

VoSviewer and Scimago Graphica were used to analyze the international geographic cooperation network in this field. A total of 25 countries have participated in scientific research on Tuina, including Asia, Europe, North America, South America, and Oceania. This involvement shows a trend of global cooperation. China is the country with the largest number of publications and the highest number of citations, indicating that the country has been active in the field of Tuina research and its achievements have been widely acknowledged. China has been involved in scientific research cooperation with many countries in the world, among which cooperation has been greatest with the United States, while cooperation with European countries such as the United Kingdom, Germany, and Switzerland has also been relatively high (Fig. 3). After China, other countries with a higher number of publications are the United States (n = 25), Germany (n = 9), Australia (n = 9), and South Korea (n = 7) (Table 2).

**Table 2:** Top 5 publications, centrality and citations of countries related to Tuina (1993–2022)

Rank	Country	Publications	Citations
1	China	284	1225
2	USA	25	289
3	Germany	9	130
4	Australia	9	110
5	South Korea	7	97

### 3.4. Distribution of institutions

**Figure 4.**
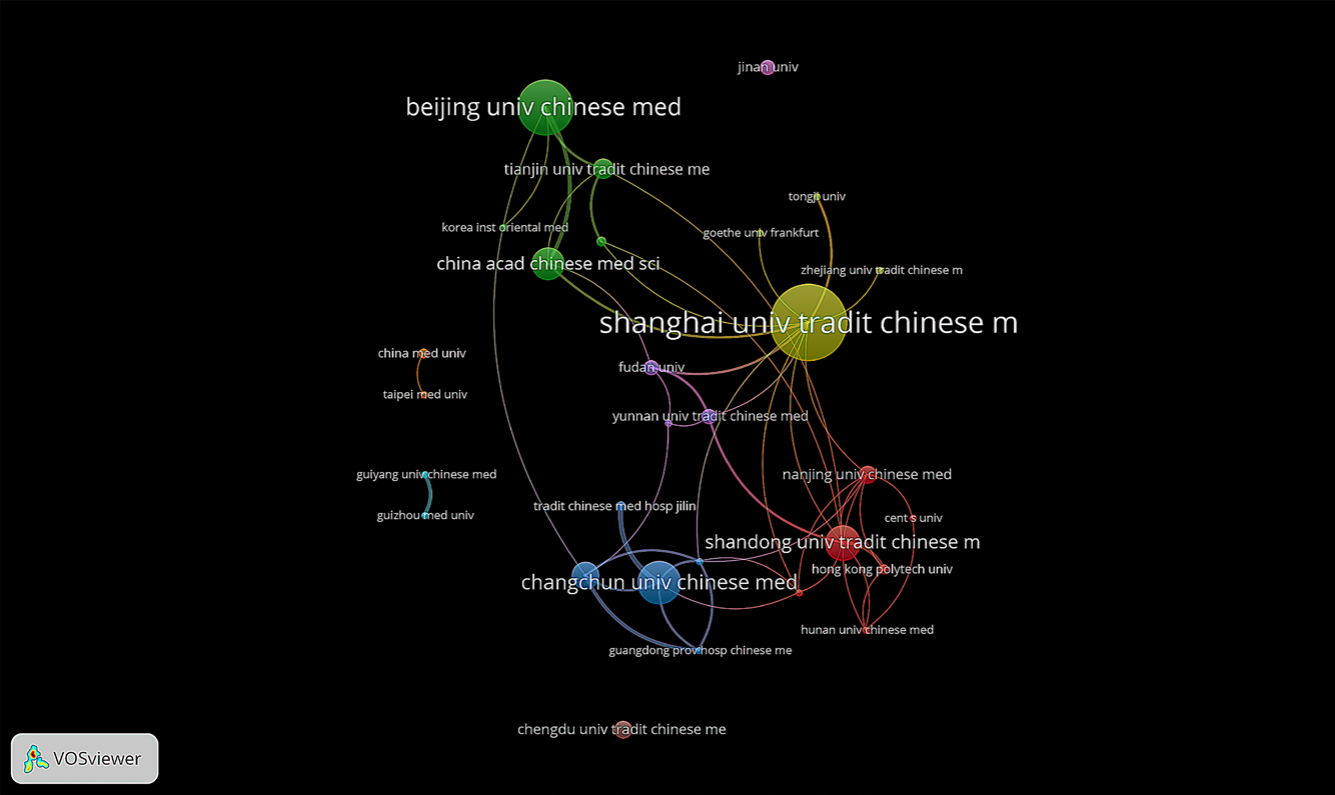
The collaboration network of institutions related to Tuina (1993–2022).

An analysis using VoSviewer of 327 institutions contributing to the research on Tuina was conducted. The Shanghai University of Chinese Medicine has published the most papers (n = 30), followed by Beijing University of Chinese Medicine (n = 22) and Changchun University of Chinese Medicine (n = 17). The top 3 institutions cited frequently are Shanghai University of Chinese Medicine (n = 238), Beijing University of Chinese Medicine (n = 166), and China Academy of Chinese Medical Sciences (n = 117) (Table 3). The institutional cooperation network diagram shows the cooperation between major institutions (Fig. 4). As can be seen in this figure, in addition to some institutions having only a small amount of cooperation and output, a cluster of cooperative institutions with major achievements and mutual cooperation has formed in the middle, in which 5 cluster clusters are present. The yellow cluster shows the cooperation between Shanghai University of Chinese Medicine, Shanghai Jiao Tong University, Tongji University, Frankfurt University, etc. The green cluster shows the cooperation between Beijing University of Chinese Medicine, Tianjin University of Chinese Medicine, Korea Medical College of Oriental Medicine, China Academy of Chinese Medical Sciences, etc. The blue cluster shows the cooperation between Changchun University of Chinese Medicine, Jilin Hospital of Traditional Chinese Medicine, Guangdong University of Chinese Medicine, etc. The red cluster shows the cooperation between Shandong University of Chinese Medicine, Nanjing University of Chinese Medicine, Hong Kong Polytechnic University and Hunan University of Chinese Medicine, etc. The purple cluster shows the cooperation between Yunnan University of Chinese Medicine and Fudan University, etc. It can be seen that although there is international cooperation in the scientific research of Tuina, it is still dominated by cooperation between Chinese institutions.

**Table 3:** Top 12 publications, centrality and citations of countries related to Tuina (1993–2022)

Rank	Institution	Publications	Citations
1	Shanghai univ tradit chinese med	30	238
2	Beijing univ chinese med	22	166
3	Changchun univ chinese med	17	18
4	Shandong univ tradit chinese med	14	45
5	China acad chinese med sci	13	117
6	Guangzhou univ chinese med	11	33
7	Tianjin univ tradit chinese med	8	42
8	Nanjing univ tradit chinese med	7	26
9	Chengdu univ tradit chinese med	7	13
10	Fudan univ	6	36
11	Yunnan univ tradit chinese med	6	20
12	Jinan univ	6	84

### 3.5. Distribution of authors

We analyzed 1157 authors who published articles on Tuina, 27 of whom published more than 3 articles. Table 4 lists the 15 authors who published the most articles. The top 5 authors included Min Fang (n = 17), Qingguang Zhu (n = 7), Zhiwei Wu (n = 6), Lingjun Kong (n = 6), and Yufeng Wang (n = 6). The most cited authors are Jianping Liu (n = 68) and Min Fang (n = 58).

**Table 4:** Top 15 publications and centrality of authors related to Tuina (1993–2022)

Rank	Author	Publications	Citation
1	Min Fang	17	58
2	Qingguang Zhu	7	24
3	Zhiwei Wu	6	11
4	Lingjun Kong	6	12
5	Yufeng Wang	6	4
6	Shuaipan Zhang	5	8
7	Qiang Tian	5	11
8	Xinghe Zhang	5	16
9	Jianping Liu	5	68
10	Xiantao Tai	4	14
11	Yanbin Cheng	4	21
12	Bo Chen	4	19
13	Hourong Wang	4	12
14	Juan Yu	4	10
15	Rusong Guo	4	9

**Figure 5.**
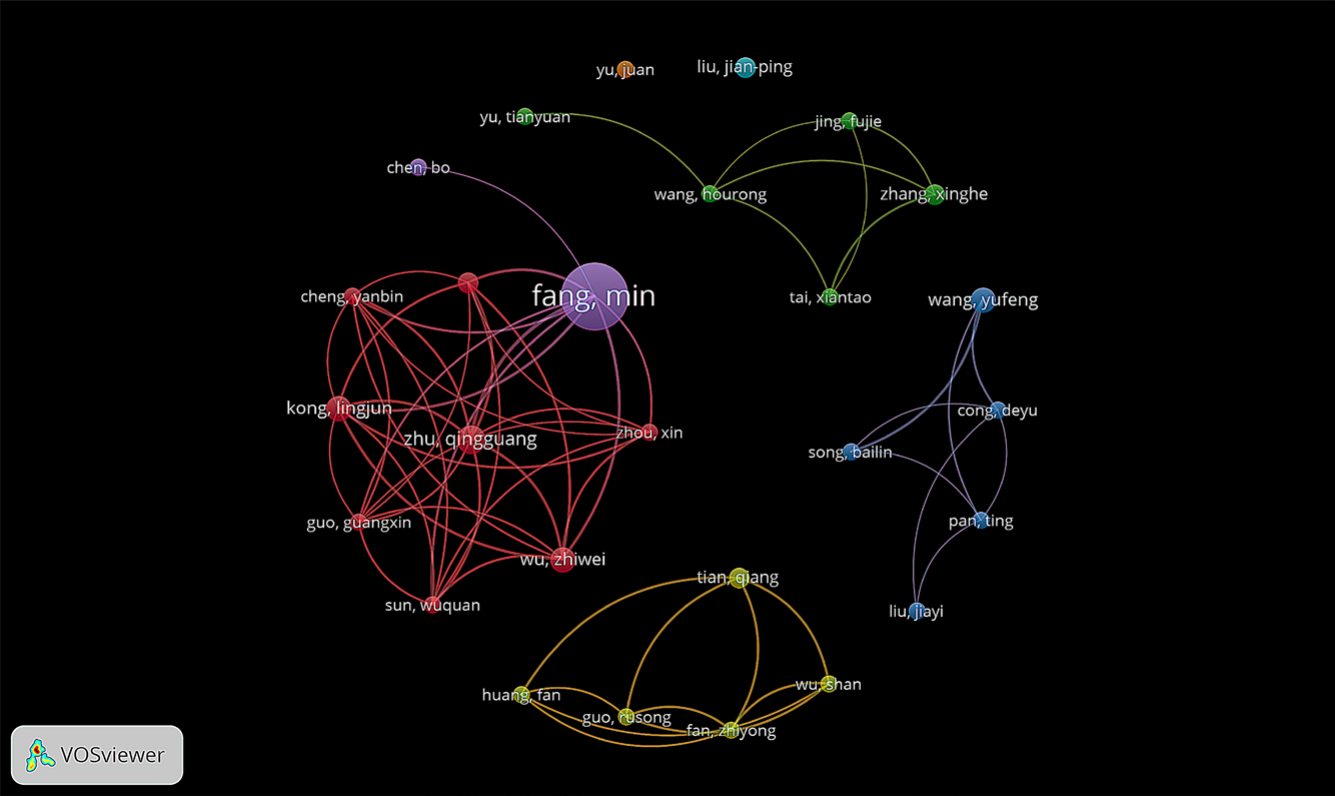
The collaboration network of authors related to Tuina (1993–2022).

The authors’ cooperative network formed 5 clusters. The purple cluster was represented by Min Fang and Bo Chen. Their research focused on evaluating the clinical efficacy of Tuina in the treatment of musculoskeletal and spinal diseases and poststroke depression.^[13–17]^ In addition, there are studies on the analgesic mechanism of Tuina,^[18,19]^ the dose-effect relationship of Tuina,^[20]^ and biomechanical study of Tuina.^[21–23]^ The red cluster has the largest number of authors, among which Zhu Qingguang, Zhiwei Wu, Linjun Kong, and others are representative authors. Their studies have a close relationship with the purple cluster. They primarily focus on evaluating the effectiveness of Tuina therapy for various conditions, including osteoarthritis, lumbar disc herniation, poststroke depression, and other diseases.^[16,17,24,25]^ Additionally, they conduct research on the mechanism of Tuina analgesia and assess the efficacy of Tuina in treating chronic fatigue syndrome.^[26]^ The purple and red clusters have recently focused on fMRI studies of Tuina therapy for osteoarthritis and poststroke depression and have investigated the mechanism of Tuina action from the perspective of neuroimaging.^[27,28]^ The representative authors in the green cluster are Xiantao Tai, Tianyuan Yu, Fujie Jing, Xinghe Zhang, and Hourong Wang. Their research has focused on pediatric Tuina. These clinical studies on pediatric Tuina paid attention to promote the development of premature infants,^[29]^ treating functional constipation and pediatric cerebral palsy.^[30,31]^ Additionally, animal experiments have been conducted to investigate the potential of Tuina in preventing neonatal ischemia and hypoxia by inhibiting the neuroinflammatory response.^[32]^ The blue cluster is represented by Yufeng Wang, Deyu Cong, Bailin Song, Ting Pan, and Jiayi Liu. Their studies focused mainly on evaluating the efficacy of Tuina in the treatment of apoplexy sequelae,^[33–36]^ diabetic peripheral neuralgia,^[37]^ cervical hypertension,^[38]^ and simple obesity.^[39]^ The representative authors of the yellow cluster are Qiang Tian, Fan Huang, Rusong Guo, Zhiyong Fan and Shan Wu. Their research has focused mainly on evaluating the clinical efficacy of Tuina on low back pain,^[2,40]^ cervical vertigo,^[41]^ lumbar disc herniation,^[42]^ and tension headache (Fig. 5).^[43]^

### 3.6. Analysis of journals

A total of 113 journals published publications concerning Tuina research. The top 10 journals are listed in Table 5. Six of the 113 journals published more than 10 articles. The “*Journal of Acupuncture and Tuina Science*” has published 80 articles, and is the most published journal, followed by “*Medicine*” (n = 49), “*Evidence-Based Complementary and Alternative Medicine*” (n = 18), “*Complementary Therapies in Medicine*” (n = 14), “*Journal of Traditional Chinese Medicine*” (n = 13), and “*Trials*” (n = 11).

**Table 5:** Top 10 publications and centrality of journals related to Tuina (1993–2022)

Rank		Journal	Publications	Impact factor (2022)	JCR partition
1	Journal of Acupuncture and Tuina Science		80	0.5	Q4
2	Medicine		49	1.6	Q3
3	Evidence-Based Complementary and Alternative Medicine		18	N/A	Q3
4	Complementary Terapies in Medicine		14	3.6	Q2
5	Journal of Traditional Chinese Medicine		13	2.6	Q3
6	Trials		11	2.5	Q4
7	World Journal of Acupuncture-Moxibustion		7	0.7	Q4
8	Journal of Alternative and Complementary Medicine		7	2.6	Q3
9	Chinese Journal of Integrative Medicine		5	2.9	Q3
10	Complementary Therapies in Clinical Practice		4	3.0	Q2

JCR = Journal Citation Reports.

**Figure 6.**
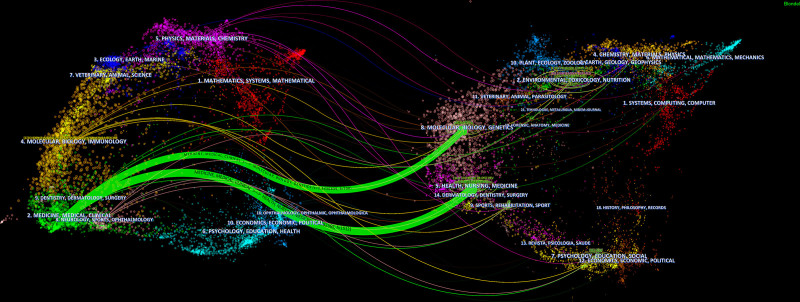
The dual-map overlays of journals in Tuina (1993–2022).

Figure 6 shows that the literature in the field of medicine, clinical medicine, and the literature in the field of molecular biology, genetics, health, nursing, and other fields have been mutually cited. This indicates that researchers not only verify the efficacy of Tuina in clinical studies, but also attempt to investigate the potential mechanism of Tuina efficacy at the molecular and genetic levels.^[44]^

### 3.7. Analysis of keywords

VoSviewer was used to analyze the 944 keywords obtained with the objective of helping to understand research trends and hot spots in the field. A total of 59 keywords with a co-occurrence frequency of not less than five were selected. The keyword with the highest co-occurrence frequency was “Tuina” (n = 83), followed by “systematic review and meta-analysis” (n = 55), “acupuncture” (n = 43), “massage” (n = 40), “protocol” (n = 34), and “randomized controlled trial” (n = 33) (Table 6).

**Table 6:** Top 10 keywords related to Tuina (1993–2022)

Rank	Keyword	Occurrences
1	Tuina	83
2	Systematic review and meta-analysis	55
3	Acupuncture	43
4	Massage	40
5	Protocol	34
6	Randomized controlled trial	33
7	Management	24
8	Efficacy	21
9	Children	18
10	Quality of life	17

**Figure 7.**
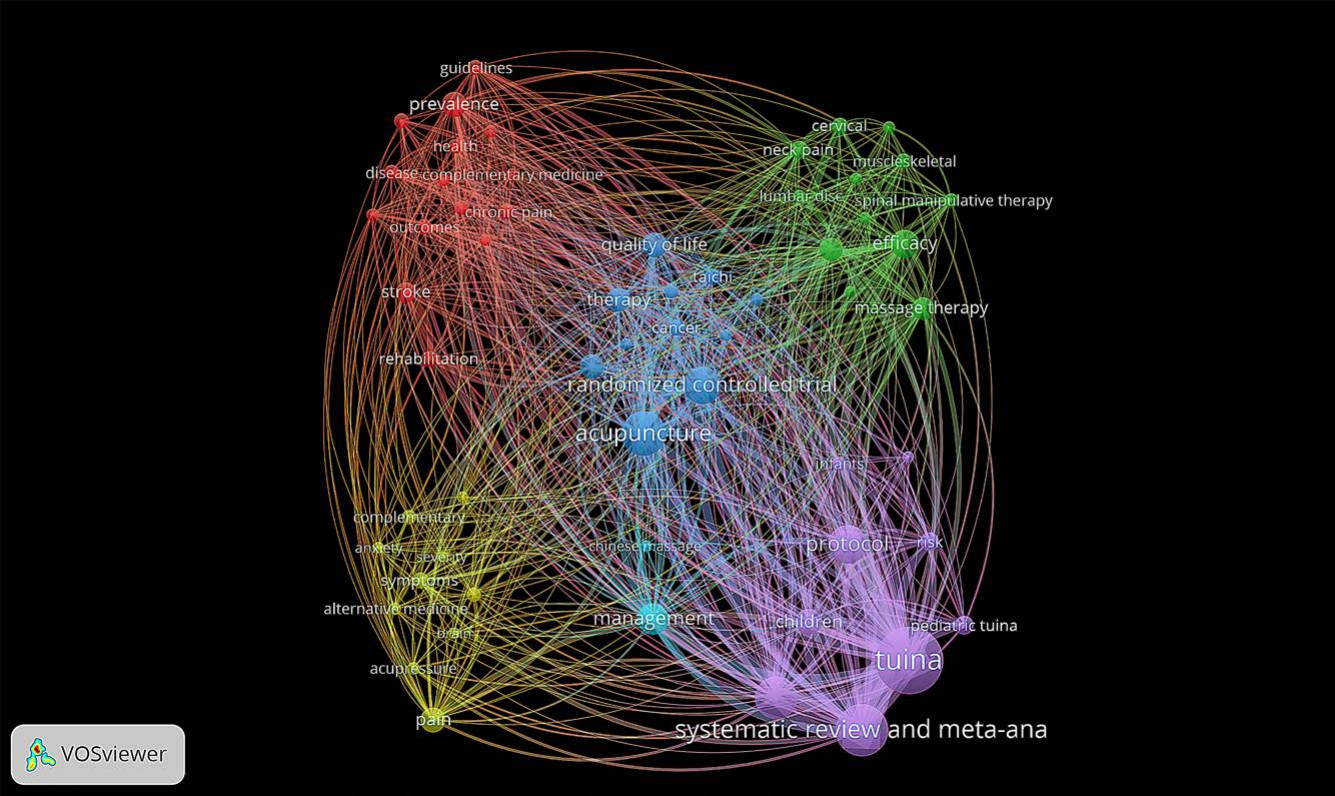
The network of co-occurrence keywords related to Tuina (1993–2022).

As shown in Figure 7, 5 clusters were formed by the analysis. The main keywords in the red cluster were “prevalence,” “stroke,” “chronic pain,” etc. The cluster-related research included the evaluation of efficacy of Tuina in the treatment of stroke sequelae^[34–36]^ and chronic pain.^[45–47]^ The main keywords in the blue cluster were “acupuncture,” “randomized controlled trial,” “quality-of-life,” “therapy,” “cancer,” “taichi,” etc. The main research related to the cluster focused on the evaluation of efficacy and social investigation of TCM comprehensive therapy including Tuina to reduce blood pressure^[48]^ and improve the quality-of-life of cancer patients.^[49–52]^ The main keywords in the green cluster were “efficacy,” “cervical,” “neck pain,” “lumbar disc,” “muscles skeleton,” “spinal therapy” etc. This cluster included the study of musculoskeletal and spinal diseases such as using Tuina for cervical spondylosis,^[53,54]^ lumbar spine disease,^[55,56]^ and tendinitis.^[57]^ The main keywords in the yellow cluster were “pain,” “complementary,” “anxiety,” “acupressure,” “severity,” etc. Related studies included the use of self-rating anxiety scales to observe the clinical efficacy of Tuina in the treatment of musculoskeletal pain^[58,59]^ and chronic fatigue syndrome^[60]^ from an emotional level. The main keywords in the purple cluster were “Tuina,” “systematic review and meta-analysis,” “protocol,” “children,” “pediatric Tuina,” “infants,” etc. Relevant studies have focused on the evaluation of the clinical efficacy of pediatric Tuina in the treatment of autism spectrum disorders,^[61]^ fever in children,^[62]^ anorexia in children,^[63]^ developmental delay in premature infants,^[29]^ functional constipation,^[30]^ enuresis,^[64]^ attention deficit and other pediatric diseases.^[65]^

**Figure 8.**
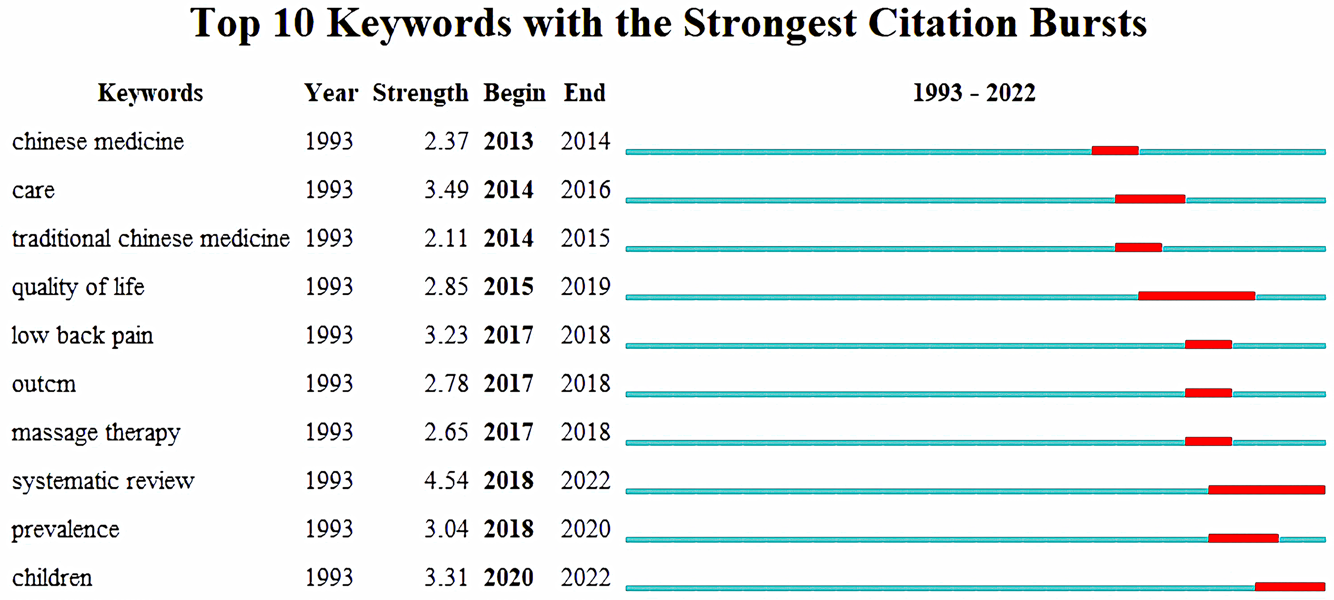
The top 10 keywords with the strongest citation bursts related to Tuina (1993–2022).

CiteSpace was used to analyze the burst keywords. Burst keywords refer to keywords that have attracted high attention within a certain period of time. “Chinese Medicine” was the earliest burst keyword, maintaining a high-intensity outbreak in 2013 to 2014, while “quality-of-life” maintained an outbreak for a long time from 2015 to 2019. “Systematic review” and “children” were the most popular keywords in the past five years (Fig. 8).

## 4. Discussion

Bibliometrics is a quantitative research method based on published and textual data, serving as a useful tool for describing the development trends in scientific fields.^[66]^ The results of bibliometric analysis include not only descriptive statistics but also network analyses of keywords, countries, journals, authors, institutions, and their relationships. It examines the frequency, relevance, centrality, and clustering of authors and textual data, which scholars often use to explore the evolution patterns of topics, publication trends, author citation networks, and other key information.^[67]^ To understand the development trends of Tuina research and provide references for scholars and clinicians in the field.

The initial publication on Tuina appeared in 1993, marking the commencement of research in this area. From 1993 to 2010, the first phase, saw a minimal annual output of scholarly articles on Tuina. The subsequent phase, from 2011 to 2016, experienced a modest rise in publication numbers compared to the earlier period. Both phases exhibited stability without significant expansion. Notably, starting in 2017, there has been a substantial annual increase in Tuina-related research publications, signaling growing interest and global recognition of the field.

Regarding geographical origins of these publications, China leads with 284 papers, significantly more than the United States, which has 25. Other countries contribute fewer than ten publications each. Figure 3 highlights a trend of national collaboration within Tuina therapy research, notably between China and the U.S., while also revealing the insufficiency of current collaborative efforts in the field and underscoring the need for intensified international cooperation.

Institutionally, Shanghai University of Traditional Chinese Medicine contributes the most to Tuina literature, followed by Beijing University of Chinese Medicine and Changchun University of Chinese Medicine. While cooperation among institutions has expanded globally, it is primarily collaboration between Chinese institutions. The potential to further strengthen international partnerships is evident.

Regarding author contributions, Min Fang leads with the highest number of publications, followed by Qingguang Zhu, Zhiwei Wu, Lingjun Kong, and Yufeng Wang. All four top scholars hail from Shanghai University of Traditional Chinese Medicine, where they primarily conduct clinical research and systematic reviews on Tuina therapy for conditions such as low back pain,^[13,24]^ knee osteoarthritis,^[14,15]^ lumbar disc herniation,^[16]^ post-stroke depression,^[17]^ chronic neck pain,^[25]^ and chronic fatigue syndrome.^[26]^ They also investigate the analgesic mechanisms of Tuina, demonstrating increased Piezo2 expression, decreased Piezo1 expression, and reduced peripheral nociceptive C fiber activity, which may explain its efficacy in alleviating abnormal pain and hyperalgesia.^[18,19]^ Additionally, the biomechanical characteristics and dose-response relationship of Tuina therapy are urgent areas of investigation. To address this, researchers have conducted mechanical testing on the “Yi Zhi Chan” manipulation, summarizing its biomechanical properties.^[21]^ They studied the effects of different intensities and durations of “Yi Zhi Chan” on popliteal artery blood flow, finding that a vertical force of 9.31 N for 10 minutes optimally improves peripheral circulation.^[20]^ Scholars from Shanghai University of Traditional Chinese Medicine, especially Min Fang, are highly productive in both basic and clinical research on Tuina therapy, accounting for their significant impact.

In terms of journals, Table 5 lists the top 10 journals that have published the most Tuina research. “Journal of Acupuncture and Tuina Science” publishes the most studies, likely due to its focus on Tuina. Most Tuina research is also published in journals related to complementary and alternative medicine, albeit in smaller quantities. This trend may be attributed to researchers’ submission habits. Combining author contributions and keyword analysis, Tuina research tends to lean towards clinical studies and favors journals that accept clinical trials. This also highlights the current deficiency in basic research in Tuina studies. Basic research is crucial for elucidating the mechanisms of Tuina and optimizing treatment protocols. Future consideration should be given to conducting relevant basic research.

Keyword analysis indicates that Tuina research is prominent in clinical trials, particularly RCTs, with further verification in systematic reviews and meta-analyses. This is significant for Tuina’s clinical application and promotion. However, there is a relative scarcity of studies on Tuina’s mechanisms and biomechanics. Future research should strengthen basic studies to investigate treatment mechanisms and optimize strategies. Tuina has been extensively studied for analgesia, especially musculoskeletal pain, and for improving quality-of-life in chronic diseases and cancer. Recent clinical research, reports, and systematic reviews on pediatric Tuina indicate its extensive clinical applications.

Recent burst keywords in Tuina therapy include “systematic review” and “children,” focusing on various pediatric diseases such as cough variant asthma,^[68]^ anorexia,^[69]^ cough,^[70]^ muscular torticollis,^[71]^ and allergic rhinitis.^[72]^ These studies suggest growing interest in pediatric Tuina therapy. Pediatric Tuina differs from adult Tuina in specific acupoint use and emphasis on gentle techniques. Despite its potential applications, significant knowledge gaps remain regarding its clinical efficacy and mechanisms, necessitating further investigation. Thus, pediatric Tuina may become a new focal point in future Tuina research.

### 4.1. Recommendations for future work

As the exploration of mechanisms and clinical application promotion of Tuina continue, research in this field is expected to gradually increase. Currently, the main institutions and authors involved in Tuina research are primarily domestic. In the future, it will be necessary to enhance international cooperative exchanges to foster development. Furthermore, as a non-pharmacological therapy, besides clarifying mechanisms and verifying clinical efficacy, the dose-response relationship and standardization of techniques in Tuina are also important topics. The research process may require the involvement of disciplines such as biomechanics, with multidisciplinary collaboration being beneficial for ensuring the scientific rigor and reliability of the research. Studies have shown that pediatric Tuina is more commonly applied in internal medicine diseases, and the research on its clinical evidence is becoming a hot topic. However, the mechanisms of action remain unclear, and there is a need for stronger foundational research in this area in the future.

## 5. Limitations

As far as we are aware, this is the first bibliometric analysis of the research status and trends of Tuina therapy. However, this study had some limitations. On one hand, the data for the study was sourced exclusively from the WoSCC database, due to limitations in the bibliometric analysis software for conducting analyses across multiple databases. However, the WoSCC database is currently regarded as the most authoritative and comprehensive academic literature database worldwide as it is also highly compatible with bibliometric analysis software,^[73]^ thereby ensuring the accuracy of analytical results. This has resulted in this database being used widely in many bibliometric analyses.^[74,75]^ However, there may be a small number of publications in other databases, such as PubMed, that have not been retrieved. On the other hand, bibliometric analysis reveals the development trends of a certain field within a specific time period and is time-sensitive. Due to changes in the number and direction of future research, it is necessary to analyze new trends based on the latest studies after a period of time.

## 6. Conclusion

The research on Tuina has been increasing year by year. Currently, the focus of Tuina research lies in clinical studies, including the treatment of skeletal muscle and spinal diseases, stroke sequelae, chronic diseases, and pediatric illnesses, with pediatric Tuina emerging as a hot topic of research. Basic research in this field is relatively scarce, and the mechanisms of action of this therapy have not yet been fully elucidated. Future efforts in this area should aim to strengthen basic research and promote cooperation among international institutions.

## Acknowledgments

The authors would like to express their gratitude to EditSprings (https://www.editsprings.cn) for the expert linguistic services provided.

## Author contributions

**Conceptualization:** Lin Gao.

**Data curation:** Lin Gao, Suyu Chen, Tianlei Gao, Liuying Yang.

**Formal analysis:** Lin Gao.

**Methodology:** Lin Gao.

**Visualization:** Tianlei Gao, Liuying Yang.

**Writing – original draft:** Lin Gao, Suyu Chen.

**Writing – review & editing:** Xiantao Tai.
